# Intimate Partner Violence in the Sub-Saharan African Immigrant Community in Chicago: A Changing Landscape

**DOI:** 10.3390/epidemiologia3030026

**Published:** 2022-07-04

**Authors:** Kathryn Wenham, Bernadette Sebar, Patricia Lee, Neil Harris, Gabrielle Campbell

**Affiliations:** 1 Public Health, School of Health and Behavioural Sciences, University of the Sunshine Coast, Sippy Downs, QLD 4556, Australia; gcampbell@usc.edu.au; 2 Public Health, School of Medicine and Dentistry, Griffith University, Southport, QLD 4222, Australia; b.sebar@griffith.edu.au (B.S.); patricia.lee@griffith.edu.au (P.L.); n.harris@griffith.edu.au (N.H.)

**Keywords:** intimate partner violence, violence against women, African immigrants

## Abstract

The challenges of conducting research on intimate partner violence (IPV) in immigrant communities means little is known about the occurrence of various forms of IPV, making it difficult to address in these populations. This research draws on data gathered in Chicago’s large and varied African immigrant communities. This research used a mixed methods approach: collection of quantitative survey data on occurrence, followed by qualitative interviews to explain the results. Missing quantitative data and contradicting qualitative responses made it difficult to draw definite conclusions on physical IPV; however, verbal abuse and controlling behaviours appear to be relatively widespread and normalised, and not always viewed as violence. Particularly with the probability of future pandemics and natural disasters, which are known to increase prevalence, it is important to raise awareness of less visible controlling behaviours and verbal abuse as forms of violence, and to implement appropriate prevention programs to minimise a concomitant rise in IPV within African immigrant communities.

## 1. Introduction

Women are disproportionately affected by intimate partner violence (IPV); however migrant and refugee women in particular are at increased risk because the contexts of vulnerability mean that many are isolated from family, experience unstable employment, economic dependence, precarious migration status, language barriers, and lack of access to suitable support services [1,2]. The COVID-19 pandemic and its associated lockdowns, social isolation, job losses, and economic turmoil saw a concurrent pandemic in intimate partner violence (IPV) across many parts of the world [3,4]. This increase in IPV was largely hidden as people were forced to stay home, and access to both formal and informal support systems for both perpetrators and victims were limited [5]. It is, therefore, important to better understand the types of IPV that occur within immigrant and refugee communities so more effective prevention efforts can be developed, especially in the event of future pandemics and natural disasters, which frequently cause an increase in IPV [6].

Research on the occurrence of IPV in immigrant communities is challenging to conduct effectively [7,8]. As a result, very little is known about this phenomenon in terms of prevalence [9]. The lack of a definitive sampling frame to allow random sampling, the sensitive nature of IPV, and unwillingness to report IPV can make it difficult to accurately estimate the prevalence of these behaviours [10,11]. As a result, little research has been conducted on the prevalence of IPV within immigrant communities, with IPV only detected if or when people seek help [10,11]. Consequently, the result is a lack of understanding of the breadth and shape of IPV within these communities. Having a clearer understanding of the types of IPV prevalent within various immigrant communities would better clarify how to plan relevant prevention programs and enable the appropriate provision of services.

Ogunsiji and Clisdell [12] conducted a literature review of IPV prevention and reduction among migrants. They concluded that interventions needed to be based on theory, grounded in the cultural context of the migrants, involve participation of stakeholders such as community leaders and members, target both survivors and perpetrators, and ground and embed strategies for evaluation. This research aims to provide data that will help to guide future interventions and identify further research needs.

The sub-Saharan African immigrant community is one of the fastest growing immigrant communities in the United States; however, relatively little is known about the state of IPV within this population [11]. More specifically, the sub-Saharan African immigrant community is a collection of diverse communities from a variety of national, ethnic, language, and religious backgrounds [13]. Similarly, their motivation for migration varies, including for economic, education, family or humanitarian reasons [13]. Despite such diversity within African immigrants, they often group themselves together, as they have in Chicago under the banner of the United African Organization. This unity is partly in recognition of the many commonalities shared by these diverse communities, as well as the strength they gain in working together to address the common challenges of living as diasporic communities in the United States. However, preliminary discussions with community leaders highlighted differences between the predominantly black sub-Saharan Africans and the predominantly Arab North Africans in terms of experiences of racism and its effects on employment, which are possible contributing factors to IPV. Therefore, this research will focus on sub-Saharan African immigrant communities. While anecdotal evidence from domestic violence service providers suggest IPV is an issue in African immigrant communities, the literature on IPV within these communities is largely qualitative in nature and addresses neither the extent of IPV within these communities, nor which types of IPV are more prevalent [11]. The literature does report that IPV can be quite extreme in some communities, sometimes resulting in homicide [10].

The literature also suggests that African women have difficulty accessing services appropriate to their needs [11,14]. Since IPV is often only revealed when people seek help, the lack of appropriate services can lead to an underestimation of IPV within these communities. Similarly, physical violence might be more evident than other forms of violence because it is easier to recognise [15], and women possibly seek help for it more frequently compared to less visible forms of abuse.

Given the gaps in the literature and the need for information to plan for more appropriate services, this research aims to document the nature and extent of IPV in African communities in Chicago. While these data were collected prior to the pandemic, the results can help to inform future research and program needs to prevent IPV in general, and during times of crises, such as pandemics and other natural disasters.

## 2. Materials and Methods

Considering the challenges of conducting prevalence research in immigrant communities, this study used mixed methods, including a survey and in-depth interviews, to address the research aims. Although prevalence studies are usually purely quantitative, the difficulties of gaining a representative and unbiased sample and potential for missing data with a sensitive topic limits the credibility of such research in immigrant communities. Therefore, a qualitative component was added to triangulate the data and gain an understanding into the findings of the survey.

IPV, for the purposes of this study, refers to violence by a man against his intimate partner (or previous partner). This study is limited to male violence against women because this type of violence is more common, more severe, and because migrant women are a more vulnerable population [2]. Both men and women were included as participants to obtain both perspectives on male violence. For the purposes of this paper, definitions of IPV follow the World Health Organization (2005) definitions [16]. The questionnaire from the WHO [16] Multi-country Study on Women’s Health and Domestic Violence Against Women formed the basis of this research. The WHO questionnaire had been tested and used in multiple African nations, providing credibility and the ability to compare data. Therefore, for this research, the definition of IPV follows that of WHO [16]. Consequently, physical IPV includes slapping, throwing something that could injure, pushing, shoving, pulling hair, hitting with a fist or something that could hurt, kicking, dragging, beating, choking, burning, or threatening with a weapon; sexual IPV includes physically forcing to have sexual intercourse, coercing sexual intercourse because she feared the consequences, or forcing her to do something sexual that she found degrading or humiliating; verbal IPV consists of insulting, belittling, humiliating, scaring, intimidating or threating to hurt her or someone she cares about; controlling behaviours include stopping or restricting her from seeing friends or family, insisting on always knowing where she is, ignoring her or acting indifferently, getting angry if she speaks to another man, being suspicious that she is unfaithful, or expecting her to request permission from the husband to seek health care; and financial violence includes two or more of the following: limiting the choice of a partner in terms of how she spends her money, requiring she hand over her earnings, refusing to allow a partner to work, taking their money or refusing to give them money when it is available for household expenses. Given the sensitive nature of the data and the safety issues related to research on IPV, the study protocol included collecting data within the context of a broader survey on health and wellbeing, in a manner that protected the identity of participants. An information and consent form explained the risks and benefits of the research and the non-compulsory nature of participation. Participants were also provided with information via a brochure produced by the researcher on support services for health, wellbeing and intimate partner violence to anyone who was invited to participate, as well as to community leaders. This ensured that if any issues arose from their participation, they were aware where they could obtain appropriate support. The research team also observed participants for signs of distress and were trained to respond appropriately to signs of distress in participants. There was also a licensed psychologist and domestic violence professional on the research team who was available for additional advice if required.

The United African Organization (UAO) supported and facilitated the collection of data for this research. The UAO consists of North African and sub-Saharan African communities. However, community consultation found that there were unique issues related to racial discrimination from the broader community that were faced by sub-Saharan Africans (versus predominantly Arabs from northern Africa, white Africans mostly from South Africa or Zimbabwe, or Africans of Indian descent). Some community members identified that certain types of discrimination, particularly around employment, could potentially contribute to IPV [17]. Additionally, education of women as a potential protective factor in IPV is inconsistent but possibly contextual [18]. While the African-born population in the US is in general one of the more highly educated groups [19], it is not clear whether this particularly relates to those from sub-Saharan Africa, where English is more widely spoken and therefore students are more likely to study in English speaking countries than their northern counterparts [20]. Therefore, this research, as part of a broader research project, was limited to the black sub-Saharan African population to better address their unique experiences [17].

### 2.1. Quantitative Methods

Inclusion criteria for the survey were being born in Africa, of African racial origin, 18 years of age or older, living in the greater Chicago metropolitan area, and having sufficient English to answer the survey or interview questions with minimal to no assistance. Participants were recruited using convenience sampling from a variety of community, social, and religious organisations, as well as through taxi-driver training classes to ensure a diversity of participants. It is not possible to estimate how many people were invited to participate given the overlap of people in different organisations and events.

A total of 342 people (men and women) completed the anonymous self-administered surveys throughout 2013. Self-administered questionnaires were chosen because research has shown a lower item non-response rate for self-administered questionnaires compared to interviewer administered questionnaire for surveys that contain sensitive questions [21]. Participants had the choice of completing the survey online via iPads provided by the researchers or via pen and paper. This choice was offered so that participants could use the method they felt most comfortable with and increase their chances of participation and of completing all the questions. Similarly, surveys were completed wherever people felt most comfortable to complete them, such as at their church or in their homes. Hard copies were collected in a large envelope to ensure anonymity. Surveys were administered to men and women separately to protect women who might have been experiencing violence. Research assistants employed to administer the survey were trained with training materials from the World Health Organization’s Multi-Country survey [22], and were supervised and supported by the principal researcher and a team member who was a licensed psychologist and a certified domestic violence professional in the state of Illinois. The research assistants were predominantly African-born postgraduate students from health- or welfare-related disciplines. This enabled participants to seek assistance from someone from the same or similar culture, but who was not a permanent community member who might be perceived as a threat in terms of privacy and anonymity. This was done to encourage participants to feel confident to participate and respond to all questions. Two research assistants were Muslim women, to provide appropriate assistance to Muslim women participants. The above processes were implemented in the survey design to build trust and promote confidentiality, because building trust and conveying confidentiality is essential to stimulate the provision of valid answers and minimise item non-response rates [23]. Trust was also built by collaborating with community leaders, explaining the study, seeking their advice, and gaining their support [8]. Of the total sample, 277 were ever-partnered men and women. This research followed the customary practice in IPV research of including only ever-partnered participants for data analysis related to IPV [16]. The definition of ever-partnered for the purposes of this research was those who had been married or in a long-term sexual relationship. The surveys used the core questions adapted from the World Health Organization’s (2005) Multi-country Study on Women’s Health and Domestic Violence Against Women [16], along with some additional demographic questions. The questions from the WHO questionnaire included questions about whether participants had experienced specific acts of violence including, but not limited to, being hit, kicked, belittled, humiliated, or had their income withheld [16]. Men were asked if they had done these things to their partner. For the purposes of this study, IPV was counted if one of the options in the relevant list on the survey had been checked for physical violence, sexual violence, verbal violence, or controlling behaviours. The criteria were less strict for financial abuse, where two items were required. This was in response to discussions with community members who identified a need for the criteria to be changed to better suit cultural norms around household economies. The surveys were analysed using SPSS (version 25; IBM, Armonk, NJ, USA) to provide descriptive statistics to examine the reported occurrence of various types of IPV within the sample.

### 2.2. Qualitative Methods

Following the survey, 18 in-depth semi-structured interviews (from half to one and a half hours in length) were conducted with community leaders and members over a two-week period. Some interviewees had also completed the survey; however, due to the strict practices to protect anonymity of those completing the survey, the research team were not aware who had completed the survey unless the interviewee volunteered that information. Twelve men and six women participated in the interviews. The higher number of men was intentional to better understand the male perspective in terms of what contributes to IPV. The interviews asked participants to describe IPV in their communities, its prevalence, and the forms that it took. Participants were then asked to comment on some of the survey findings around the occurrence and types of IPV.

In addition to meeting the above inclusion criteria, interview participants were purposively selected by having sufficient social networks and community involvement to provide rich and diverse interview data on the topic. This included religious and community leaders and general community members who had extensive social networks within their communities.

In contrast to the surveys, interviewers asked indirect questions to satisfy a communication preference within many African cultural groups. Interview participants were recruited from a variety of places (e.g., community groups, religious organisations, and workplaces) to increase sample diversity, and to ensure different perspectives and contextual factors [24]. Interviews were recorded with permission and transcribed.

The transcripts were first read to gain familiarity, then thematic analysis was applied in both a concept-driven and data-driven manner [25]. First, data were coded according to the main categories of IPV (concept-driven): physical, sexual, verbal, controlling behaviours, and financial abuse [16,26]. This fulfilled the requirement of unidimensionality, with each category or theme covering only one concept [25]. Within those main categories, the data were coded in a data-driven manner. As themes emerged from the data, mutually exclusive subcategories or subthemes were identified [25]. These subcategories or subthemes related to the issues raised within the responses. As the analysis progressed, the subthemes were questioned repeatedly to ensure they were accurate, mutually exclusive, contextually relevant, and reflective of the data [25,26]. In this process, some subthemes were combined or split into two as new concepts emerged or it became evident that some subthemes were really referring to the same thing. Such flexibility and continued refinement in data analysis has been recommended to ensure that there is space for fresh observations and changes of direction within the analysis [26]. Several rounds of analysis and consideration of alternative themes and interpretations confirmed the choice of themes and subthemes [26].

### 2.3. Ethical Considerations

The study was approved by the Institutional Review Board (or Ethics Committee) of The University of Illinois at Chicago (protocol code #2012-0743, approved 4 January 2013) and the Human Research Ethics Committee of Griffith University (PBH/03/13/HREC, approved 31 January 2013). Informed consent was gained from survey and interview participants. No identifying information was required, but when this was given, the transcripts were de-identified to maintain confidentiality. Pseudonyms are used to report findings, and country of origin is not reported because it is potentially identifying. Contextual factors were also identified from the interviews and noted to aid with analysis. Measures to support and protect participants are outlined in earlier sections.

## 3. Results

A total of 277 ever-partnered people completed the survey. Four respondents (0.01%) who did not clearly specify their gender were excluded from the analysis. Of the remaining 274 participants, 159 (58%) were men and 114 (42%) were women. Response rates were difficult to calculate because sometimes people had already been recruited via another venue. Where it was possible to calculate response rates, they were between 10% and 90%, with the most common rates between 40% and 80%. More men than women completed the survey. This was partly due to the essential need to protect the identity of participating women from their intimate partners. When a woman’s identity could not be protected by conducting the survey in an area private from men, data were not collected. A higher number of male participants could have influenced the results, given that men are more likely to deny violence [27].

Most participants were in the 30–39-year age group, followed by 40–49, and then 20–29. Participants reported high educational achievement, with more than half (54.6%) having completed either a four-year college degree or postgraduate education. Only 2.8% had achieved a primary school education or less, with the remainder (42.6%) having completed high school or a two-year college degree. Of these ever-partnered respondents, 75.3% were from West Africa and 21.5% were from East Africa, with the remainder from Central and Southern Africa. Most participants (76.1%) were from urban areas in Africa, and about two thirds (67.5%) had been in the United States for ten years or more. People had mostly migrated for economic or education reasons (62.6%), or to join a spouse or family (23.6%). Only 13.8% (*n* = 35) had migrated to the United States for humanitarian reasons. There were significant gender differences in the reason people migrated. More men (76.4%) than women (44.1%) had migrated for education or economic purposes, and more women (46.1%) than men (7.4%) had migrated to join their spouse or other family. More men (16.2%) than women (9.8%) had migrated for humanitarian or refugee reasons. Christianity was the predominant religion (88.1%), followed by Islam (7.5%). Fewer than 5% identified with a religion other than Christianity or Islam. Despite relatively high levels of educational achievement, occupational status levels were relatively low, with 35.2% in professional jobs, 13.0% in skilled occupations, 36.4% in semi-skilled occupations, and 15.4% in unskilled jobs.

Comparisons with the U.S. Census Bureau data for the African-born population in the Chicago statistical area were made to gauge whether the sample was representative of the broader population. The sex ratio for the survey sample was not significantly different (X^2^ (1, *n* = 338) = 2.35; *p* = 0.125); however, there were significantly more married people in the survey sample, and the survey participants were significantly more educated than the census data (X^2^ (4, *n* = 313) = 33.93; *p* = 0.001) [28,29]. However, the census data were for all African-born people, whereas the sample was limited to those born in sub-Saharan Africa. It is not clear whether this difference might account for the differences in marital status and educational status. However, the geographical spread of region of origin was similar to data from a survey by Wilson [30] on African immigrants in Chicago, with the majority of participants being from West Africa, followed by East Africa, with only a small proportion from Central or Southern Africa.

### 3.1. Survey Results: Occurrence of IPV in Ever-Partnered Respondents

#### 3.1.1. Women’s Self-Reported Experience of IPV

Women’s self-reported experience of violence by their intimate partners is shown in Table 1. In terms of lifetime experience of abuse from an intimate partner, 22.2% of female respondents reported physical abuse, and 13.4% reported sexual abuse. Higher rates were reported for verbal abuse (27.8%), controlling behaviour (25%), and financial abuse (46.4%). In contrast, only one participant (0.9%) reported past 12-month violence (physical and verbal). Past 12-month violence was not measured for controlling behaviours or financial abuse.

As is common in surveys on IPV, many participants did not answer the questions related to violence, with missing data rates of 36.8% (*n* = 42) for physical violence, 41.2% (*n* = 47) for sexual violence, 36.8% (*n* = 42) for verbal abuse, 29.8% (*n* = 34) for controlling behaviours, and 50.9% (*n* = 58) for financial abuse for the total sample of ever-partnered women (*n* = 114). The higher rate of missing data for financial abuse is possibly due to the financial abuse questions being towards the end of the questionnaire. Additionally, some people might not have responded to questions about 12-month prevalence because of the sensitivity of the question or for fear of consequences. These high rates of missing data possibly reflect participants not being comfortable to report, which possibly suggests the rates could be higher than the data indicates.

#### 3.1.2. Men’s Self-Reported Perpetration of IPV

Men’s self-reported perpetration of IPV is displayed in Table 2. A total of 6.4% of ever-partnered men reported that they had perpetrated behaviours suggestive of physical IPV in their lifetime. Similarly, 8.1% reported perpetrating behaviours indicative of sexual IPV. In contrast, 19.8% reported behaviours related to verbal IPV, 28.4% reported controlling behaviours, and 17.3% reported perpetrating two or more behaviours related to financial abuse. Compared to lifetime prevalence, men’s reports of current prevalence was considerably lower: 0.6% (*n* = 1) for verbal and sexual IPV, 0% for physical IPV, and not measured for controlling behaviours or financial abuse. Missing data rates for the total sample of ever-partnered men (*n* = 159) were lower than for women: 21.4% (*n* = 34) for physical violence, 22.6% (*n* = 36) for sexual violence, 20.8% (*n* = 33) for verbal abuse, 15.7% (*n* = 25) for controlling behaviours, and 38.4% (*n* = 61) for financial abuse.

As is common in studies of IPV, men tended to report lower rates of perpetration of abuse than women reported experiencing abuse. Exceptions to this were controlling behaviours and current sexual violence, which men reported at a slightly higher rate than women. Differences between the self-reported rates of physical IPV experienced by women and perpetrated by men were significantly different (X^2^ (1, *n* = 205) = 10.20; *p* = 0.001), with women reporting higher rates than men. Similarly, results showed a significant difference between the reported rates of financial abuse (X^2^ (1, *n* = 154) = 14.98; *p* < 0.001), with women again reporting at a higher rate.

### 3.2. Interview Findings

Many interviewees stated that they believed that despite it being acceptable in their countries of origin, physical IPV was not widespread in the community, as evidenced in the following quotes:

‘No, not in our community. The violence, especially within the families, no, that is not there.’

(William)

‘But taken to the next level of violence, it’s really rare in the African community, at least in the community that I know.’

(Beatrice)

The perception of low levels of physical IPV were commonly attributed to fear of the legal system,

‘The law. Oh yeah. Over there the culture, the financial, all that kind of stuff is different. Here the law if you hit a woman, you are already charged, you are automatically in violation. So you know the consequences, you cannot do that’

(Abel)

While many participants thought it was not common because of domestic violence laws in the US, they were realistic in recognising that it did exist,

‘I don’t think that it is necessarily pronounced in this particular community. I would not necessarily say it’s so pronounced, but does it exist? Absolutely, it does exist. There are situations of abuse’.

(Joseph)

The acceptability of physical IPV varied depending on ethnic background. For example, one participant noted,

‘One of our cultural things is that the man never hits a woman. It’s a shame. It’s a disgrace’.

(Beatrice)

In contrast, someone from a different national and ethnic background described physical IPV in their country of origin in as acceptable:

‘based on our culture you can beat the wife. It’s okay.’

(Edward)

There were clear discrepancies surrounding interviewee perceptions of the occurrence of physical IPV. Such discrepancies could possibly be attributable to cultural backgrounds, as noted above, or migration status. As one participant noted, lack of a valid visa can entrap women in abusive relationships because of a fear of deportation,

‘I don’t have papers and he is a citizen, and he can help me and let me just accept the situation and don’t want to report it because, do I really want to deal with the police when I don’t have my papers?’

(Sulayman)

Similarly, while some people disclosed violence, it is possible that some of the missing data were attributable to fear of reporting. As one respondent noted in the interviews,

‘If they’re dodging the questions, they are a little bit afraid of telling you the truth’.

(Emmanuel)

#### 3.2.1. Physical Violence

Although many participants stated that physical IPV was not widespread in their communities, a few noted that when it did happen, it could be quite severe, sometimes resulting in homicide. This was a reflection of a number of high-profile intimate partner homicides that had occurred, particularly within one ethnic group from Nigeria. This had stimulated a lot of debate within African immigrant communities, and the ensuing discussion on social media was the subject of research by Kalunta-Crumpton [31]. The following quote is from a West African participant:

‘We’ve had a lot of African men kill their wives. We’ve had a lot of it. They’re common in Texas and the rest. You don’t just wake up in a day and do that’.

(Emmanuel)

A quote from an East African participant echoed a similar issue:

‘They go into drinking, and I have a couple of them already in prison because they killed their wives’.

(Edward)

These participants were from vastly different ethnic backgrounds, demonstrating that this is not just linked to one ethnic group; however, further research would be required to determine if it is more prevalent in particular groups. Whatever the case, it is important to note that, if left unchecked, IPV has been known to escalate to homicide within the communities with which the above participants identified. This feature is not restricted to African immigrant communities. Research has shown that controlling behaviours are a risk factor for intimate partner homicide [32].

Some participants noted that they thought IPV had become less common back in Africa.

‘When we were little our uncles and maybe our fathers, they did it. But for a long time I have not seen anybody beating his wife’.

(Kingsley)

It was not clear from their statement whether IPV has become more hidden or less prevalent. In contrast, some participants described how the normalisation of IPV in the societies they came from led to people not talking about it.

‘That pattern is okay back home, you get what I mean. So you feel like that’s just one of those things you need to face. So, even though it’s happening, they wouldn’t bring it out’.

(Flore)

Many participants believed IPV in general was an issue, but for the most part African immigrants do not endorse it.

‘Now, does it happen? Probably, it does. Do some of the women report it? Definitely. Do some women just want to keep their home in peace? Yeah, … I believe it’s happening, but it’s not being glorified’.

(Mary)

All participants emphasised that the rates of physical violence were lower in the United States than where they came from because community members feared the United States legal system.

‘So it is not a common thing, both here and there. Here it is because of the law’.

(Kingsley)

Someone from a country where physical IPV was quite common and accepted stated:

‘Well, I cannot hit her. The rule in United States is different and you’re going to go to jail. The consequence is you are going to lose your job, you are afraid to do that’.

(Abel)

This is only exacerbated by the fear of deportation,

‘They don’t believe in [calling] the law enforcement in our community because of this fear of deportation’.

(Sulayman)

Although this fear possibly suppresses people talking about IPV when it happens, it seems that this fear also deters perpetrators of physical IPV because there is clearer evidence of physical IPV (e.g., bruises, welts) than of less visible forms such as verbal abuse or controlling behaviours. In less formal conversations with community members, the fear of the legal system discouraging physical IPV was a common theme. Another participant described how the legal system prevented him from enacting physical violence on his ex-wife,

‘I wish I hit my wife for the way she acted and making me mad and angry. But I can’t because the rule in United States is different’.

(Abel)

#### 3.2.2. Sexual Violence

Many participants did not comment on the prevalence of sexual IPV within the community; however, those who did explained that sexual obligations of the wife were normalised, preventing many people from recognising sexual abuse. Two participants described how sexual IPV could be common due to the belief in matrimonial obligation and the expectation that the wife is there to please her husband.

‘You find yourself doing something that if you had a chance, you wouldn’t because again you are caught under … your matrimonial obligation. If you don’t, he will go out (to seek sex outside the marriage). So, yes, sexual violence, you feel compelled to let him have his way even when it is not okay with you, because you feel like saying no is violating your matrimonial principle. But, hey, that is violence if I don’t want it’.

(Flore)

‘… sexual violence, sometimes we have. We are not considered that’s sexual violence between partners. He’s the man and you have to—because we are here to please him. So, he’s the man. I have to say yes regardless’.

(Rita)

Both of these participants suggested that sexual violence would be under-recognised and under-reported for this reason.

#### 3.2.3. Verbal Violence

Participants identified verbal and emotional abuse as relatively common,

‘Verbal and emotional are the highest number of abuse that people face’.

(Kingsley)

However, they also identified that it is likely not reported because many people do not identify it as abuse,

‘They will not even report (verbal abuse) and they don’t even really even identify that this is actually abuse’.

(Beatrice)

#### 3.2.4. Controlling Behaviours

Controlling behaviours by husbands over wives were often described as the norm in African relationships. This was often viewed as culturally or social sanctioned (if not expected) to the point where people spoke about it quite openly in interviews, as well as in the community, and with less shame than the other types of IPV. One woman described the general attitude of men in the following way:

‘Yes, the mentality is still there that I can control the woman as I want, I can do this, I can do that as I want’.

(Hannah)

This same participant described in more detail how women adapting to the host nation could exacerbate control issues extending to further conflict and potentially other forms of violence.

‘Most of the [African] men, they think they can control their wives anyhow, “Whether you like it or not, I’m your husband.” But it doesn’t work that way in this country. This country is 50-50. So maybe when their wife comes and they started saying that, “No, no, no. It’s supposed be 50-50. We are supposed to have equal rights,” they start fighting. They don’t have happy home because he wants to have full control and the woman doesn’t want to accept that. So, it affects most, most’.

(Hannah)

All the participants recognised that men frequently regarded control over women as their right or expectation as a man. However, only one participant considered this as an acceptable view.

#### 3.2.5. Financial Abuse

Financial abuse was rarely raised in interviews. Participants sometimes provided examples of financial abuse in response to questions about stress rather than questions about abuse because they tended to describe financial control as more of a trigger for conflict rather than identify it as an abuse in itself. When financial control was spoken about, participants noted that Africans tended to view the breadwinner as having earned the right to control the finances, normalising behaviours that can be interpreted as financial abuse. While, previously, the main breadwinner had usually been the man, that often shifted to women upon migration, creating changes in expectations.

‘But there is that sense of being an African man you control everything, whatever you say is right, and that creates a sense if the woman in the house is bringing in some money and she is trying to do things with the money, then that is a problem for us because I am the man, I’m supposed to tell you what to do with the money‘(laughs a lot).

(Justin)

Only one example was provided in direct response to questions about financial abuse,

‘Sometimes the money the men should have been giving to the wife (for the household), he will be spending it for the girlfriend or whoever that he’s having the affair with outside’

(Emmanuel)

Financial abuse was sometimes recognised by participants in the context of marital infidelity, rather than in the excessive control of financial affairs.

## 4. Discussion

In comparison with similar studies on comparable populations, women’s lifetime prevalence of experiencing physical IPV (22%) was at the lower end of the spectrum when compared to the WHO Multi-country Study (13–61%) [16], and the African sites within the WHO Multi-country Study (30.6% to 48.7%). Similarly, the lifetime prevalence of women being slapped, pushed, or shoved by an intimate partner in United States was 30.3% [33]. However, 36.8% of female ever-partnered participants in the current survey did not answer the question on physical IPV. Although the questionnaire was offered online to minimise missing data [34], many participants elected to complete the questionnaire via pen and paper, which may have increased the rate of missing data on IPV questions. Missing survey data cannot be assumed to be missing at random [35]. This rate of physical IPV could be higher if women refused to answer because they did not want to disclose violence; however, it is not possible to ascertain the reasons for non-response to the questions on IPV. While missing data could lead us to conclude that the occurrence of physical IPV is higher, most interviewees tended to agree that physical violence, while occurring within their communities, was not widespread. This fits with a study by Vaughn et al. [36], which found lower levels of physical IPV among African immigrants compared to U.S.-born Americans and immigrants from Asia and Latin America. However, African-born migrants had higher levels of physical IPV than European-born immigrants to the U.S. [36]. Taken on balance between the survey, interviews, and existing data, it would appear likely that the occurrence of physical IPV is not at either extreme end of the spectrum, but probably sits somewhere in between. However, it is not possible to provide a definitive figure. It possibly differs between various subgroups, but further research is required. Although there is a risk of stigmatisation, epidemiological data on diverse groups could help to tailor prevention efforts. A few interviewees claimed that a fear of the legal system was suppressing the perpetration of physical IPV. This is supported by research that also highlights fear and avoidance of the legal system surrounding IPV [14,37]. The impact that legal issues could have on people’s immigration status could also intensify this fear [14,37].

Interviewees did suggest that when physical IPV did occur, it could be serious, sometimes escalating to homicide. This recognition of serious IPV was primarily a reflection of several high-profile intimate partner homicides in a particular African ethnic group in the U.S. in the years preceding this research [10], although a few other cases of intimate partner homicides were also noted.

In contrast to the contradicting and inconclusive results regarding physical IPV, the results on controlling behaviours were more consistent. While higher numbers of ever-partnered women (25%) reported experiencing controlling behaviours, there was a lower percentage of missing data (29.8%). Additionally, while men usually under-report IPV compared to women [38], they reported a higher rate of perpetrating controlling behaviours (28.4%) than women reported experiencing, and the amount of missing data was also lower, at 15.7%. This suggests a possibility that controlling behaviours are more common and there are fewer barriers to reporting, such as lower risk of legal repercussions or social shame. In keeping with this premise, the interviewees stressed that men’s controlling behaviours towards their spouse were common and viewed as normal. Indeed, men could be using it to meet their masculine ideal of men as dominant decision makers within their family [39,40]. Such normalisation also means that many would not recognise or label it as a form of IPV. However, controlling behaviours can be as much or more detrimental to a woman’s health and wellbeing than other types of IPV [41,42,43]. According to a study of West African immigrants in Australia, controlling behaviours and emotional abuse were not viewed as violence, which supports the findings in this research [15]. It is also well documented that controlling behaviours are often a precursor for physical IPV [44] or intimate partner homicide [32]. Therefore, severe or fatal IPV in the presence of high rates of controlling behaviours and in the absence of high rates of physical IPV fits with this pattern, and suggests that controlling behaviours need to be addressed. For these reasons, it could be important to raise awareness within the community regarding the nature and outcomes of controlling behaviours, how to recognise them, and how to seek help.

Given the extent of missing data on financial abuse and the different perceptions of financial control reported within these communities, it is difficult to draw conclusions about the occurrence of financial abuse. However, given that those who did respond reported high occurrence, and the role of financial abuse in trapping women in violent relationships, research in this area is imperative.

Although it appears possible that strong legal frameworks in the U.S. might be pushing down the prevalence of physical violence, it is also possible that this might be transforming to under-reporting of physical IPV and to perpetration of less visible forms of IPV such as controlling behaviours, verbal, and financial abuse, which were reported at higher rates. These can be more difficult to verify in victims, and so may be a preferred alternative for those who are afraid of legal consequences. The normalised nature, particularly of controlling behaviours, means these actions may not be recognised by many people within the community as forms of violence that are both damaging to mental health and possible precursors to more extreme violence.

### Limitations

The lack of a sampling frame prevents random sampling in this research, which can leave the research open to sampling bias. The research was designed to minimise sampling bias by recruiting from a variety of sources, comparing demographic information from the sample to the census data and by conducting in-depth interviews to verify the picture of IPV within the community. Comparisons with census data found the sample to be somewhat representative, although participants were a little more highly educated and were limited to those who spoke English well or very well. Attempts to provide a French version of the questionnaire stopped after inconsistencies were noted in the translation process.

Given the sensitive and shameful nature of IPV, the illegality of IPV (particularly physical violence), and the insecure migration status of many within the community, under-reporting is likely, particularly for physical IPV. To encourage participants to feel comfortable responding to questions about sensitive issues, the research used anonymous online (with an option of pen and paper) surveys with no identifying data required (21]. Given the likelihood of under-reporting in IPV surveys, questionnaire data were compared to interview data to try to clarify the patterns of IPV. In addition, it was not just the numbers that were taken into account, but also the acceptability. For example, many participants commented that physical violence was unacceptable, while others noted that controlling behaviours were considered normal. This was reflected in the number of participants who reported controlling behaviours in the questionnaire.

In addition, this research considered the sub-Saharan African immigrant community as a whole. Despite community consultation highlighting that many sub-Saharan African migrants identify many commonalities between groups, the situation could (and likely does) vary for different ethnic or national groups, as well as immigration status and reason for migration, within that broader community. Data on ethnic or national backgrounds was not collected in this research because it is potentially identifiable, which raised ethical concerns. While epidemiological data on various groups (national, ethnic, reason for migration, or immigration status) would help to better address IPV in these diverse communities, smaller numbers could compromise statistical analysis and risk stigmatising any groups that have a higher prevalence.

Finally, data for this research is from 2013. While it is close to ten years since these data were gathered, there are still gaps in the literature around IPV in African immigrant communities. For example, a systematic review on IPV in racial and ethnic minorities noted that larger sample sizes are needed in this area (the largest sample size they found was 114) [45]. Community leaders concur that the issue of IPV still exists and needs to be addressed, and that attitudes have changed little in this time. They recommended that this research was still valuable. This is supported by research in Sudanese communities in Australia, which found that adults tended to hold on to traditional cultural norms and practices [46]. It is also supported by more recent research into the experience of Nigerian immigrants to the United States, which found that controlling behaviours were one of the most common types of IPV [47]. Qualitative research with African immigrants by Corley and Sabri [48] also noted that IPV, including controlling behaviours, is an issue that needs to be addressed.

While time has passed since these data were gathered, more recent research suggests this is an ongoing issue. However, the more recent research was qualitative in nature [48], or from a service providers’ perspective [47]. This research continues to fill a gap in the literature, as it is useful to guide researchers and program designers regarding potential factors that need to be further considered in relation to IPV in African migrant communities. For example, rates of financial abuse appear high, but limitations of the study mean these might not be completely accurate. However, it does suggest that this is an area in need of further research, particularly as measures for financial abuse have been developed in recent years [49]. Rates of IPV have increased globally as a result of the COVID-19 pandemic, while at the same time, services have been restricted [5]. Therefore, continued research in this area is important, and this research highlights areas that require further research.

## 5. Conclusions

It is probable that the nature of IPV within the African immigrant community in Chicago leans towards less visible forms of IPV, such as controlling behaviours and verbal and financial abuse. This suggests that further research and raising awareness of these lesser known but frequent forms of IPV is needed so that the community can recognise them and respond with appropriate prevention initiatives. Many community leaders and organisations expressed an openness to addressing issues of IPV in their communities, and thus could be important avenues to reach the community. It is important that the community has access to the resources that it needs to appropriately address IPV, and to help men to express masculinity in more gender-equitable ways. Prevention efforts against IPV are necessary to try to reduce IPV levels in general, and prevent spikes occurring during times of crisis, such as during pandemics and other natural disasters.

## Figures and Tables

**Table 1:** Prevalence of ever-partnered women experiencing intimate partner violence (IPV).

Type of IPV ^1^	Lifetime Prevalence ^2^		Last 12 Months
	Yes	No	Yes ^3^
Physical *n* = 72	22.2% (16)	77.8% (56)	0.9% (1)
Sexual *n* = 67	13.4% (9)	86.6% (58)	0% (0)
Verbal *n* = 72	27.8% (20)	72.2% (52)	0.9% (1)
Controlling *n* = 80	25.0% (20)	75.0% (60)	NA
Financial *n* = 56	46.4% (26)	53.6% (30)	NA

^1^*n* = 114 (Valid n changes for each type of IPV and relates only to the yes/no responses for lifetime prevalence). ^2^ Numbers and percentages for lifetime prevalence are from all ever-partnered women who responded to these questions. ^3^ Only those who had answered yes to lifetime prevalence were invited to respond regarding 12-month prevalence. This resulted in very few responses for past-year prevalence. NA = Not applicable.

**Table 2:** Prevalence of ever-partnered men perpetrating IPV.

Type of IPV ^1^	Lifetime Prevalence ^2^		Last 12 Months ^3^
	Yes	No	Yes
Physical *n* = 125	6.4% (8)	93.6% (117)	0% (0)
Sexual *n* = 123	8.1% (10)	91.9% (113)	0.6% (1)
Verbal *n* = 126	19.8% (25)	80.2% (101)	0.6% (1)
Controlling *n* = 134	28.4% (38)	71.6% (96)	NA
Financial *n* = 98	17.3% (17)	82.7 (81)	NA

^1^*n* = 159 (Valid n changes for each type of IPV and relates only to the yes/no responses for lifetime prevalence). ^2^ Numbers and percentages for lifetime prevalence are from all ever-partnered women who responded to these questions. ^3^ Only those who had answered yes to lifetime prevalence were invited to respond regarding 12-month prevalence. This resulted in very few responses for past-year prevalence. NA = Not applicable.

## Data Availability

The data presented in this study are available on request from the corresponding author. The data are not publicly available due to ethical restrictions regarding participant privacy.
